# Clinical value of conventional magnetic resonance imaging combined with diffusion-weighted imaging in predicting pelvic lymph node metastasis of cervical cancer

**DOI:** 10.3389/fonc.2023.1267598

**Published:** 2023-12-22

**Authors:** Leilei Fan, Liguo Ma, Rennan Ling, Xiaojing Guo, Haili Li, Degui Yang, Zhesi Lian

**Affiliations:** ^1^ Department of Gynecology, Shenzhen People’s Hospital, The Second Clinical Medical College of Jinan University, The First Affiliated Hospital of Southern University of Science and Technology, Shenzhen, China; ^2^ Department of Radiology, Shenzhen People’s Hospital, The Second Clinical Medical College of Jinan University, The First Affiliated Hospital of Southern University of Science and Technology, Shenzhen, China; ^3^ Department of Pathology, Shenzhen People’s Hospital, The Second Clinical Medical College of Jinan University, The First Affiliated Hospital of Southern University of Science and Technology, Shenzhen, China; ^4^ Department of Public Health, Tufts University School of Medicine, Boston, MA, United States

**Keywords:** cervical cancer, pelvic lymph node metastasis, MRI diffusion-weighted imaging, apparent diffusion coefficient, predictive factor

## Abstract

**Background:**

In cervical cancer (CC), the involvement of pelvis lymph nodes is a crucial factor for patients’ outcome. We aimed to investigate the value of conventional magnetic resonance imaging (MRI) combined with diffusion-weighted imaging (DWI) and the apparent diffusion coefficient (ADC) in predicting CC pelvic lymph node metastasis (PLNM).

**Methods:**

This retrospective study included CC patients who received surgical treatments. Surgical pathology results served as the gold standard for investigating the diagnostic performance of conventional MRI combined with DWI. We analyzed the association between tumor ADC and PLNM, as well as other pathological factors. The areas under the receiver operating characteristic curves (AUCs) for ADC in assessing PLNM and pathological factors were evaluated, and optimal cut-off points were obtained.

**Results:**

A total of 261 CC patients were analyzed. PLNM patients had significantly lower tumor ADC (0.829 ± 0.144×10^-3^mm^2^/s vs. 1.064 ± 0.345×10^-3^mm^2^/s, p<0.0001), than non-PLNM CC. The agreement between conventional MRI combined with DWI and pathological results on PLNM diagnosis was substantial (Kappa=0.7031, p<0.0001), with 76% sensitivity, 94.31% specificity, and 90.8% accuracy. The AUC of tumor ADC was 0.703, and the optimal cut-off was 0.95×10^-3^ mm^2^/s. In multivariate analysis model 1, tumor ADC<0.95×10^-3^mm^2^/s was significantly associated with PLNM (OR, 2.83; 95%CI, 1.08–7.43; p= 0.0346) after adjusting for age and pathological risk factors. In multivariate analysis model 2, tumor ADC<0.95×10^-3^mm^2^/s (OR, 4.00; 95%CI, 1.61–9.89; p=0.0027), age<35 years old (OR, 2.93; 95%CI, 1.04–8.30; p=0.0428), increased tumor diameter on MRI (OR, 2.17; 95%CI, 1.18–3.99; p=0.0128), vaginal vault involvement on MRI (OR, 2; 95%CI, 1.002–3.99; p=0.0494) were independent predictors for PLNM. Tumor ADC<0.95×10^-3^mm^2^/s was significantly associated with higher risk of tumor diameter ≥4cm (OR, 2.60; 95%CI, 1.43–4.73; p=0.0017), muscular layer infiltration >1/2 (OR, 5.46; 95%CI, 3.19–9.34; p<0.0001), vaginal vault involvement (OR, 2.25; 95%CI, 1.28–3.96; p=0.0051), and lymphovascular space involvement (OR, 3.81; 95%CI, 2.19–6.63; p<0.0001).

**Conclusion:**

Conventional MRI combined with DWI had a good diagnostic performance in detecting PLNM. The tumor ADC value in PLNM patients was significantly lower than that in non-PLNM patients. Tumor ADC <0.95×10^-3^mm^2^/s, age <35 years old, increased tumor diameter on MRI, vaginal vault involvement on MRI were independent predictors for PLNM.

## 1 Introduction

In 2020, approximately 604,000 new cervical cancer (CC) cases and 342,000 deaths were reported globally, with 85% of cases and 90% of deaths occurring in low- and middle-income countries (1). While CC screening and the human papillomavirus (HPV) vaccine have led to a decrease in incidence and mortality in many parts of the world, these rates have increased over the past two decades without achieving similar reductions in developing countries (2–4). The persistent global disease burden of CC underscores the crucial need for early detection and tertiary prevention, especially in resource-limited settings. A recent report showed that pelvic lymph node (PLN) involvement reduced the 5-year survival rate for CC from 91.2% to 59.8% (5), significantly affecting the prognosis and shifting treatment from surgery to chemoradiation or brachytherapy (6–8). Guidelines recommend radiological imaging (computed tomography [CT], magnetic resonance imaging [MRI], and positron emission tomography [PET]/CT) for CC at stage IB2 or greater, particularly for nodal or extrapelvic tumor evaluation (7). Although the use of PET/CT for advanced CC is increasing, its routine application in early stages is hampered by high costs, limited availability, and radiation exposure in many countries (9). Considering these factors, MRI is the preferred imaging modality by many gynecological surgeons for staging CC, screening for surgical candidacy, and preoperative pelvic and abdominal anatomical assessments. The high-contrast resolution of MRI enables the distinction between cancerous and normal soft tissues. However, conventional MRI may not be able to discriminate between enlarged inflammatory lymph nodes and metastatic nodes, and size (short axis ≥10 mm) is still the main cross-sectional imaging criterion for identifying metastatic nodes (10). Compared with conventional MRI, diffusion-weighted imaging (DWI) can enhance the visibility of minor abnormalities and better characterize tissues and their pathological changes by providing higher sensitivity to the diffusion of water molecules in tissues (11). Furthermore, it can be quantified using apparent diffusion coefficient (ADC). ADC maps can assist in the diagnosis of local malignancy or nodal metastasis, because ADC is lower in malignancies than in normal tissues (12). DWI with ADC has been recognized as a helpful marker for lymph node follow-up after chemoradiotherapy when patients are not suitable for surgery, because changes in ADC values correlate with treatment response (13). Moreover, ADC is an independent predictor of parametrial and/or lymph node involvement (14–16). However, researchers have also observed overlaps in ADC values between benign and metastatic lymph nodes, with no standard ADC threshold. Additionally, the sample sizes of several DWI and ADC studies are limited. Further studies are required before its widespread implementation. This study aimed to investigate the value of conventional MRI combined with DWI functional imaging and ADC in predicting PLN metastasis (PLNM) in patients with CC.

## 2 Materials and methods

### 2.1 Study design and patient selection

This was a retrospective study of patients with CC who underwent surgical treatment at our hospital from December 2012 to January 2020. The inclusion criteria were as follows: (1) patients with clinical staging ranging from IA2 to IIA2 (prior to any imaging examination) assessed by at least two experienced gynecologists through a comprehensive gynecological examination at our hospital, (2) patients who underwent radical CC surgery with pelvic lymphadenectomy at our hospital, (3) patients who underwent preoperative pelvic and abdominal MRI examinations at our hospital, and (4) patients with histopathologically confirmed CC by our hospital’s pathology department. The exclusion criteria were as follows: (1) patients lacking the DWI sequence in their MRI examination, (2) patients who received neoadjuvant chemotherapy before surgery, and (3) patients with a combination of two or more types of pelvic malignant cancer. A total of 261 patients were included in this study.

### 2.2 Measurement and outcomes

The cancer diagnosis was confirmed by surgical resection. Pathological results were used as the gold standard to investigate the diagnostic performance of conventional MRI and DWI. The clinical data of the patients were collected from the electronic health medical system by clinicians. The pelvic and abdominal regions were scanned using an 8-channel phased array coil with a Siemens Magnetom SKYRA 3.0-T MR System or Magnetom Avanto 1.5-T MR System. The patients were placed in the supine position with their heads entering first for the examination. MRI sequences included the following: (1) sagittal turbo-spin-echo (TSE)-T2-weighted imaging (T2WI) sequence (echo time [TE], 80–110 ms; repetition time [TR], 3000–5000 ms; matrix, 512×512; field of view [FOV], 240–250×240–250 mm; flip angle, 150°; slice thickness, 4 mm; interslice gap, 1 mm; total number of slices, 20); (2) high-resolution axial TSE-T2WI sequence (thin-section oblique axial scanning was performed perpendicular to the longitudinal axis of the uterus in the sagittal plane, with a slice thickness of 3 mm and with the rest of the parameters similar to [1]); (3) sagittal, axial, and fat-suppressed TSE-T1-weighted imaging (T1WI) sequences (TE, 10–15 ms; TR, 500 ms; matrix, 320×320; FOV, 240×240–320 mm; flip angle, 180°; slice thickness, 5 mm; interslice gap, 1.75 mm; total number of slices, 20); and (4) axial DWI imaging (single-shot spin echo-echo planar imaging sequence with diffusion weighting at b-values of 0, 500, and 1000 s/mm²). For ADC map, the raw data were processed using diffusion software on a workstation to generate an ADC map. This was compared with conventional plain scans and DWI images at a b-value of 1000 s/mm^2^ for the primary tumor and metastatic PLNs to obtain measurements from the central region of the largest slice. A region of interest of approximately 50 mm² (avoiding necrotic cystic areas, calcifications, hemorrhagic regions, and the cervical canal area) was placed on the solid portion of the tumor and metastatic lymph nodes and measured thrice in different orientations for each lesion. The average ADC values of the tumors were analyzed in this study. A blinded review of all MRI images was performed by an experienced radiologist using the Picture Archiving and Communication System. MRI staging was performed in accordance with the 2018 International Federation of Gynecology and Obstetrics guidelines. The MRI diagnosis was primarily based on conventional MRI and DWI. While the ADC maps and values were reviewed, they mainly served to give radiologists a general impression of high or low values, functioning as auxiliary diagnostic tools rather than being subjected to strict threshold criteria.

The following parameters were collected using conventional abdominopelvic MRI and DWI:

1. Tumor size and ADC value: three orthogonal planes on T2WI were obtained. The tumor exhibited intermediate-to-high signal intensity on T2WI and could be differentiated from the slightly low signal intensity of the normal cervical tissue. The maximum tumor dimensions were measured as anteroposterior×transverse×cephalocaudal, with the largest diameter selected. Lesions exhibited a markedly high signal on DWI and a low signal on ADC maps, where the ADC measurements were taken.
2. Cervical infiltration depth: categorized into muscular layer infiltration <1/2 (mucosal layer and superficial muscle layer) and muscular layer infiltration >1/2 (deep muscle layer and full thickness).
3. Vagina and fornix involvement and location: primarily observed in the sagittal plane, indicated by the localized disappearance of the fornix or interruption of the low signal in the upper two-thirds of the vagina.
4. Other involvements: parametrial (lesion involving the full thickness of the cervix with complete disruption or disappearance of the low-signal serosa layer, extending into the surrounding fat), adnexa, bladder, rectum, and distant metastasis.
5. PLN groups: Based on anatomical locations on axial T2WI sequence, PLNs were divided into 10 groups (left and right common iliac, external iliac, internal iliac, obturator, and deep femoral lymph node groups). Enlarged lymph nodes identified on the T1WI axial sequences were also considered. A lymph node with a short axis diameter ≥10 mm, with signal characteristics on T2WI, DWI, and ADC maps consistent with the primary tumor, was recorded as a metastatic lymph node. Lymph nodes with a short axis diameter <10 mm but exhibiting signal intensity similar to that of the primary tumor, internal necrosis, disappearance of the lymph node hilum, ring enhancement, irregular or spiculated margins, and an oval, round, or lobulated shape were also considered metastatic. The ADC values of the metastatic lymph nodes were measured. Because the scan did not reach the para-aortic lymph node region, these lymph nodes were not included in this study.

### 2.3 Statistical analyses

Clinical data were analyzed as means with standard deviations for continuous variables and frequencies with proportions for categorical variables. Categorical and continuous variables of the different groups were compared using the chi-squared and t-tests, respectively. The sensitivity, specificity, positive predictive value (PPV), negative predictive value (NPV), and accuracy were calculated to evaluate diagnostic performance. Cohen’s kappa coefficient was calculated using the inter-rater agreement test: almost perfect (0.81–1.00), substantial (0.61–0.80), moderate (0.41–0.60), fair (0.21–0.40), none to slight (0.01–0.20), and no agreement (≤0). The areas under the receiver operating characteristic (ROC) curves (AUCs) for conventional MRI combined with DWI and ADC values were analyzed. The optimal cut-off points were obtained using the Youden’s J index and balancing between the sensitivity and specificity. Mixed-effects models were used to address the correlation between lymph node samples, since multiple nodes were evaluated in the same patients. Logistic regression was used for the univariate and multivariate analyses. Statistical significance was defined as a two-sided p-value < 0.05. SAS 9.4 was used to perform all statistical analyses.

## 3 Results

### 3.1 Patient basic characteristics

In total, 261 patients with CC were included in this study. The patient basic information is presented in **Table 1**. Fifty patients were diagnosed with PLNM, and 211 with non-PLNM CC. The average age at diagnosis was 50.23 years, with average ages of 21.81 years at first intercourse and 23.27 at first childbirth. Most patients (92.34%) had one sexual partner. In total, 222 patients underwent HPV testing. Among them, 111 (50%) were HPV 16 (+), 27 (12.16%) were HPV 18 (+), and 19 (8.56%) were HPV (–). There were four cancer types: 201, squamous cell carcinomas; 45, adenocarcinomas; 9, adenosquamous carcinomas; and 6, special pathological types of CC. Other tumor characteristics are shown in **Table 1**. The average tumor ADC value was 1.019 ± 0.329×10^–3^ mm^2^/s. Among the patients who had positive PLNM results on MRI, 43 had ADC value of the abnormal lymph nodes available. The average ADC value of abnormal lymph nodes was 0.897 ± 0.290×10^–3^ mm^2^/s.

**Table 1:** Patients characteristics.

	All Patients (n=261)	PLNM (-) (n=211)	PLNM (+) (n=50)	P Value
**Age**	50.23 ± 10.76	50.38 ± 10.64	49.60 ± 11.34	0.78
<35 years old	22 (8.43)	14 (6.64)	8 (16)	0.0321
**Age at 1st intercourse (n=213)**	21.81 ± 2.72	21.71 ± 2.70	22.19 ± 2.76	0.3088
<20 years old	45 (21.13)	39 (22.81)	6 (14.29)	0.0697
No. of sexual partners				
1	241 (92.34)	191 (90.52)	50 (100)	0.0768
2	11 (4.21)	11 (5.21)	0 (0)	
≥2	9 (3.45)	9 (4.27)	0 (0)	
**Age at first childbirth (n=160)**	23.27 ± 3.47	23.37 ± 3.58	22.88 ± 3.04	0.4703
<20 years old	21 (13.13)	17 (13.39)	4 (12.12)	0.0768
**No. of pregnancies**	3.87 ± 1.85	3.88 ± 1.87	3.80 ± 1.76	0.78
0	2 (0.77)	2 (0.95)	0 (0)	0.6406
1	17 (6.51)	12 (5.69)	5 (10)	
2	44 (16.86)	36 (17.06)	8 (16)	
>2	198 (75.86)	161 (76.3)	37 (74)	
**No. of deliveries**	2.39 ± 1.45	2.43 ± 1.52	2.26 ± 1.14	0.388
0	9 (3.45)	9 (4.27)	0 (0)	0.2947
1	64 (24.52)	51 (24.17)	13 (26)	
2	90 (34.48)	69 (32.7)	21 (42)	
>2	98 (37.55)	82 (38.86)	16 (32)	
**No. of vaginal delivery**	2.25 ± 1.56	2.29 ± 1.62	2.10 ± 1.27	0.3725
0	24 (9.2)	22 (10.43)	2 (4)	0.2867
1	69 (26.44)	52 (24.64)	17 (34)	
2	71 (27.2)	56 (26.54)	15 (30)	
>2	97 (37.16)	81 (38.39)	16 (32)	
**No. of caesarean section**	27 (10.34)	21 (9.95)	6 (12)	0.6691
HPV Types (n=222)				
HPV 16 (+)	111 (50)	85 (47.49)	26 (60.47)	0.1264
HPV 18 (+)	27 (12.16)	23 (12.85)	4 (9.3)	0.5228
HPV 31,33,45,52,58 (+)	35 (15.77)	26 (14.53)	9 (20.93)	0.3007
Other Types (including unknown)	60 (27.03)	54 (30.17)	6 (13.95)	0.0316
HPV (-)	19 (8.56)	15 (8.38)	4 (9.3)	0.8461
Cancer Types				
Squamous cell carcinoma	201 (77.01)	161 (76.3)	40 (80)	0.9129
Adenocarcinoma	45 (17.24)	38 (18.01)	7 (14)	
Adenosquamous carcinoma	9 (3.45)	7 (3.32)	2 (4)	
Special pathological types	6 (2.3)	5 (2.37)	1 (2)	
**Tumor diameter (cm, long axis)**	2.98 ± 1.31	2.81 ± 1.27	3.71 ± 1.23	<0.0001
<2cm	50 (19.16)	47 (22.27)	3 (6)	<0.0001
2cm≤d<4cm	141 (54.02)	121 (57.35)	20 (40)	
≥4cm	70 (26.82)	43 (20.38)	27 (54)	
Degree of differentiation (n=253)				
Well	10 (3.95)	10 (4.9)	0 (0)	0.216
Moderately	202 (79.84)	163 (79.9)	39 (79.59)	
Poorly	41 (16.21)	31 (15.2)	10 (20.41)	
Muscular layer infiltration				
>1/2	153 (58.62)	109 (51.66)	44 (88)	<0.0001
Involvement				
Vaginal vault	77 (29.5)	57 (27.01)	20 (40)	0.0702
lymphovascular space	99 (37.93)	58 (27.49)	41 (82)	<0.0001
Neural	9 (3.45)	6 (2.84)	3 (6)	0.2714
Parametrial	5 (1.92)	1 (0.47)	4 (8)	0.0052
LN diameter (mm, short axis, n=89) *	8.29 ± 4.80	6.41 ± 2.35	8.74 ± 5.13	0.007
**ADC (×10^-3^ mm²/s, Tumor)**	1.019 ± 0.329	1.064 ± 0.345	0.829 ± 0.144	<0.0001
**ADC (×10^-3^ mm²/s, Lymph node, n=43) ^†^**	0.897 ± 0.290	1.119 ± 0.412	0.829 ± 0.206	0.0565

Values are mean ± SD or n (%). PLNM, pelvic lymph node metastasis; LN, lymph node; ADC, DWI image-derived apparent diffusion coefficient.

*Among lymph nodes in patients who have positive PLNM results on MRI and had size data available;

^†^ Among patients who have positive PLNM results on MRI. The lowest ADC site is analyzed if there are multiple PLNM sites in one patient.

(-): Indicates a negative test result for HPV infection. (+): Indicates a positive test result for the presence of the HPV type specified.

The PLNM group had significantly more younger patients (age <35 years) (16% vs. 6.64%, p=0.0321), larger tumor diameter (3.71 ± 1.23 cm vs. 2.81 ± 1.27 cm, p<0.0001), more patients with muscular layer infiltration >1/2 (88% vs. 51.66%, p<0.0001), more lymphovascular space involvement (82% vs. 27.49%, p<0.0001), more parametrial involvement (8% vs. 0.47%, p=0.0052), and lower tumor ADC (0.829 ± 0.144×10^–3^ mm^2^/s vs. 1.064 ± 0.345×10^–3^ mm^2^/s, p<0.0001) compared with the non-PLNM group.

### 3.2 Diagnostic performance of conventional abdominopelvic magnetic resonance imaging combined with diffusion-weighted imaging

The agreement between conventional MRI combined with DWI and pathological results for PLNM diagnosis is shown in **Table 2**. Thirty-eight patients with PLNM and 199 patients without PLNM were correctly diagnosed using MRI and DWI. The agreement was substantial (kappa, 0.7031, p<0.0001; sensitivity, 76%; specificity, 94.31%; PPV, 76%; NPV, 94.31%; and accuracy, 90.8%).

**Table 2:** Agreement between MRI and Pathological Results on PLNM.

		Pathological results		Total
		PLNM (+)	PLNM (-)	
**MRI**	PLNM (+)	38	12	50
	PLNM (-)	12	199	211
	Total	50	211	261

PLNM, pelvic lymph node metastasis; MRI, magnetic resonance imaging.

Kappa, 0.7031(p<0.0001), Sensitivity, 76%, Specificity, 94.31%, PPV, 76%, NPV, 94.31%, Accuracy, 90.8%.

(-): Indicates a negative finding, reflecting the absence of pelvic lymph node metastasis, as confirmed by pathological results or MRI. (+): Indicates a positive finding for pelvic lymph node metastasis, as confirmed by pathological results or MRI.

**Figure 1:**
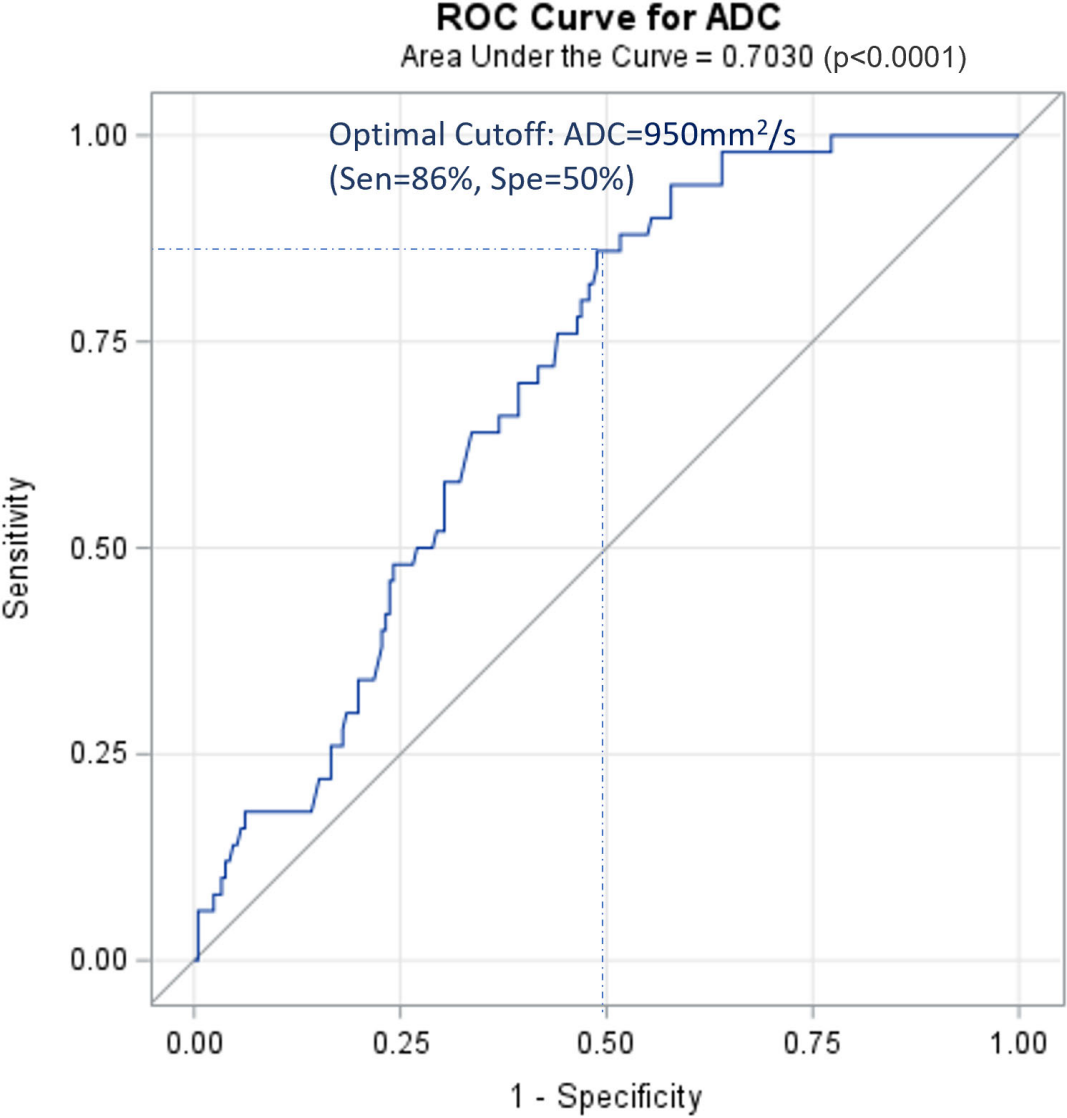
ROC curve for ADC of cervical cancer primary lesions on PLNM diagnosis. ROC, receiver operating characteristic curve; PLNM, pelvic lymph node metastasis; ADC, DWI image-derived apparent diffusion coefficient.

**Figure 1** shows the ROC curve for tumor ADC in PLNM diagnosis. The AUC was 0.703. The optimal cut-off was ADC=0.95×10^–3^ mm^2^/s, with a sensitivity of 86% and a specificity of 50%.

### 3.3 Associations between risk factors and pelvic lymph node metastasis

**Table 3** shows the associations between the risk factors and PLNM. In the univariate analysis, age <35 years, increased tumor diameter, muscular layer infiltration >1/2, lymphovascular space involvement, parametrial involvement, vaginal vault involvement (on MRI), and tumor ADC were significantly associated with PLNM. Two multivariate analyses were also performed. Clinical factor (age <35 years), pathological factors, and ADC value were included in model 1: age <35 years (odds ratio [OR], 3.98; 95% confidence interval [CI], 1.01–15.73), lymphovascular space involvement (OR, 9.39; 95% CI, 3.79–23.30), and ADC <0.95×10^–3^mm^2^/s (OR, 2.83; 95% CI, 1.08–7.43) remained significantly associated with PLNM. Clinical factor (age <35 years) and MRI results were included in model 2: age <35 years (OR, 2.93; 95% CI, 1.04–8.30), tumor diameter (OR, 2.17; 95% CI, 1.18–3.99), vaginal vault involvement (OR, 2; 95% CI, 1.002–3.99), and ADC <0.95×10^–3^ mm^2^/s (OR, 4.00; 95% CI, 1.61–9.89) were independent predictors of PLNM.

**Table 3:** Associations between Risk Factors and PLNM.

Clinical Factors	Univariate Analysis		Multivariate Analysis			
			Model 1		Model 2	
	OR (95%CI)	P value	OR (95%CI)	P value	OR (95%CI)	P value
Age <35 years	2.68 (1.06-6.80)	0.0378	3.98 (1.01-15.73)	0.049	2.93 (1.04-8.30)	0.0428
Age of initial sexual intercourse <20 years	0.56 (0.22-1.44)	0.2302				
Age at first childbirth <20 years	0.89 (0.28-2.86)	0.8481				
Number of pregnancies	0.89 (0.56-1.43)	0.6332				
Number of deliveries	0.998 (0.7-1.423)	0.9906				
Number of vaginal deliveries	0.97 (0.71-1.32)	0.854				
Pathological Factors						
Tumor diameter (long axis) *	3.44 (2.01-5.89)	<0.0001	1.91 (0.94-3.88)	0.0733		
Poorly differentiation	1.43 (0.65-3.16)	0.3755	0.91 (0.35-2.37)	0.8445		
Muscular layer infiltration>1/2	6.86 (2.81-16.79)	<0.0001	2.51 (0.82-7.68)	0.1077		
Vaginal vault involvement	1.80 (0.95-3.42)	0.0725	1.06 (0.48-2.35)	0.8792		
Lymphovascular space involvement	12.02 (5.50-26.27)	<0.0001	9.39 (3.79-23.30)	<0.0001		
Neural involvement	2.18 (0.53-9.04)	0.2824	0.43 (0.09-2.06)	0.29		
Parametrial involvement	18.26 (1.99-167.20)	0.0101	2.55 (0.24-27.60)	0.4401		
MRI Factors^†^						
Tumor diameter (long axis) *	2.98 (1.77-5.00)	<0.0001			2.17 (1.18-3.99)	0.0128
Muscular layer infiltration≥1/2	4.21 (1.71-10.35)	0.0017			1.39 (0.48-3.97)	0.5439
Vaginal vault involvement	2.89 (1.54-5.42)	0.001			2 (1.002-3.99)	0.0494
ADC^‡^	0.996 (0.995-0.998)	<0.0001				
ADC<0.95×10^-3^ mm²/s	6.20 (2.67-14.41)	<0.0001	2.83 (1.08-7.43)	0.0346	4.00 (1.61-9.89)	0.0027

PLNM, pelvic lymph node metastasis; ADC, DWI image-derived apparent diffusion coefficient.

*Analyzed as ordinal categorical variable (<2cm,2cm≤d<4cm, ≥4cm).

^†^ All the value or diagnosis are obtained from MRI exam.

^‡^Analyzed as continuous variable.

### 3.4 Using conventional magnetic resonance imaging combined with diffusion-weighted imaging to evaluate lymph node metastasis

**Figure 2:**
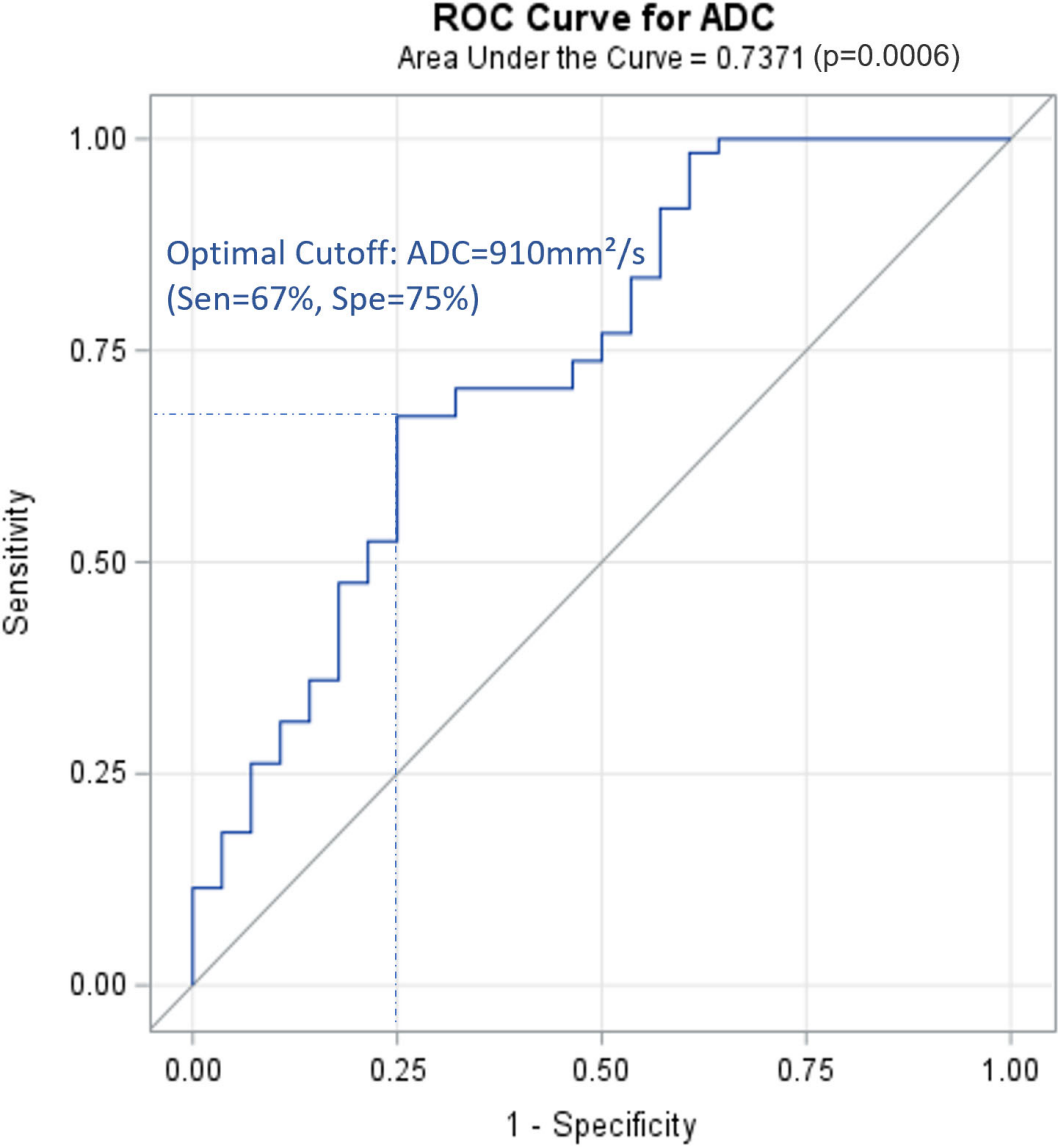
ROC curve for ADC of positive lymph nodes on PLNM diagnosis (89 lymph node data from 43 patients with positive PLNM results on MRI). ROC, receiver operating characteristic curve; PLNM, pelvic lymph node metastasis; ADC, DWI image-derived apparent diffusion coefficient.

A total of 9562 lymph nodes were resected during surgery. Characteristics of the lymph nodes are presented in **Table 4**. Conventional MRI combined with DWI correctly diagnosed 69 positive and 2476 negative lymph nodes, with substantial agreement with the pathology results (kappa, 0.67; sensitivity, 68.3%; specificity, 98.7%). Among the patients who had positive lymph node results on MRI and DWI, the ADC values of these lymph nodes were available for 89 lymph nodes in 43 patients. A ROC curve was generated for these patients (**Figure 2**). The AUC was 0.7371, and the optimal cut-off was ADC=0.91×10^–3^ mm²/s, with a sensitivity of 67% and a specificity of 75%. When addressing the correlation between lymph node samples from the clustered data using mixed-effects models, the ADC remained significantly associated with lymph node metastasis, with p=0.0002 for the ADC value (as a continuous variable) and p=0.017 for the ADC level (as a categorical variable with cut-off at 0.91×10^–3^ mm²/s).

**Table 4:** Description of lymph nodes of each site (n=9562).

PLN Regions		Total No. of Case (Total No. of LN removed)	Pathology PLN (+)	MRI PLN (+)	True Positive	False Positive	False Negative	True Negative	Sensitivity	Specificity
Total PLN*		261 (9562)	102	101	69	32	33	2476	68.3%	98.7%
Common Iliac LN	L	231 (742)	6	5	4	1	2	224	86.7%	99.4%
	R	252 (851)	10	10	9	1	1	223		
External iliac LN	L	237 (877)	9	10	5	5	4	204	56.5%	98.9%
	R	226 (830)	9	13	8	5	1	239		
Internal iliac LN	L	217 (767)	9	10	6	4	3	221	69.0%	98.1%
	R	234 (859)	19	19	14	5	5	241		
Obturator LN	L	255 (1578)	12	12	8	4	4	212	69.0%	97.7%
	R	252 (1533)	19	17	12	5	7	210		
Deep femoral LN	L	228 (770)	6	4	3	1	3	228	60.0%	98.7%
	R	225 (755)	3	1	0	1	3	221		

PLN, pelvic lymph node; LN, lymph node; L, left; R, right.

*Agreement between MRI and pathological results on positive lymph node Sites: Kappa, 0.67 (p<0.0001), PPV, 67.6%, NPV, 98.7%, Accuracy, 97.5%.

### 3.5 Associations between tumor apparent diffusion coefficient value and pathological factors

**Figure 3:**
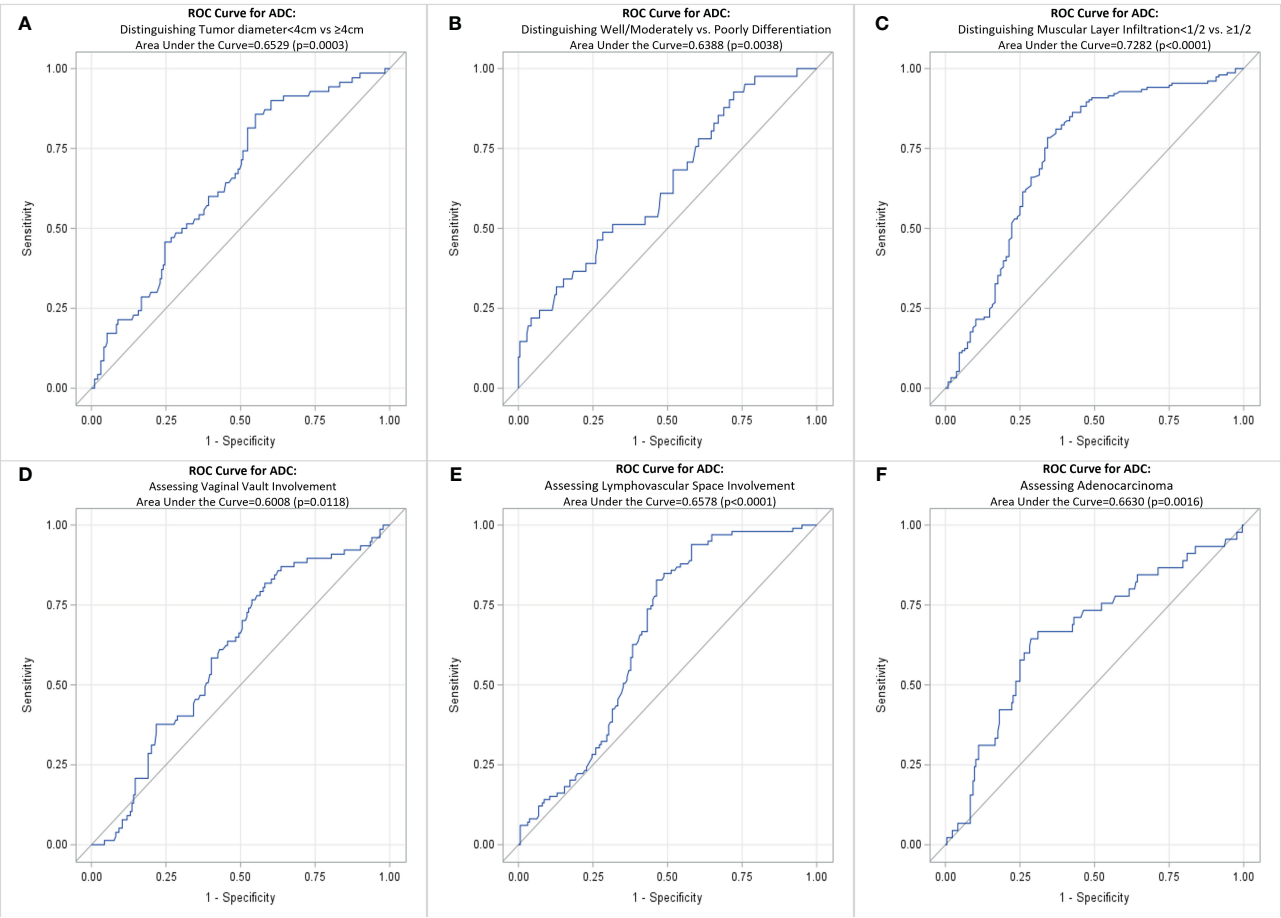
ROC curve for ADC of cervical cancer primary lesions on assessing various pathological factors of cervical cancer. **(A)** Distinguishing Tumor diameter<4cm vs ≥4cm; **(B)** Distinguishing Well/Moderately vs. Poorly Differentiation; **(C)** Distinguishing Muscular Layer Infiltration<1/2 vs. >1/2; **(D)** Assessing Vaginal Vault Involvement; **(E)** Assessing Lymphovascular Space Involvement; **(F)** Assessing Adenocarcinoma. ROC, receiver operating characteristic curve; ADC, DWI image-derived apparent diffusion coefficient.

**Table 5** shows the average tumor ADC value of different size, differentiation, infiltration, involvement, and cancer type groups. The presence of these pathological factors—including tumor diameter ≥4 cm, poorly differentiation, muscular layer infiltration >1/2, vaginal vault involvement, lymphovascular space involvement, neural involvement, and parametrial involvement—was associated with significantly lower average ADC values. However, no significant differences in average ADC values were observed between the presence and absence of squamous cell carcinoma and adenosquamous carcinoma. **Table 6**; **Figure 3** present the ROC curves and the AUCs for ADC value in evaluating the significant pathological factors identified (neural and parametrial involvement were not included owing to insufficient positive cases). The optimal cut-off for the ADC values for each pathological factor ranged from 0.939×10^–3^ mm^2^/s to 0.978×10^–3^ mm^2^/s.

**Table 5:** Average ADC value of different pathological factors groups.

Pathological Factors	Average ADC Value (×10^-3^mm^2^/s)	P Value
Tumor diameter<4cm	1.066 ± 0.345	<0.0001
Tumor diameter≥4cm	0.99 ± 0.242	
Well/Moderately differentiation	1.048 ± 0.336	0.0002
Poorly differentiation	0.879 ± 0.238	
Muscular layer infiltration<1/2	1.178 ± 0.364	<0.0001
Muscular layer infiltration>1/2	0.907 ± 0.249	
Vaginal vault involvement (-)	1.053 ± 0.344	0.0052
Vaginal vault involvement	0.938 ± 0.277	
Lymphovascular space involvement (-)	1.104 ± 0.367	<0.0001
Lymphovascular space involvement	0.881 ± 0.189	
Neural involvement (-)	1.026 ± 0.333	0.0006
Neural involvement	0.828 ± 0.116	
Parametrial involvement (-)	1.021 ± 0.332	0.0028
Parametrial involvement	0.905 ± 0.053	
Squamous cell carcinoma (-)	1.088 ± 0.359	0.0655
Squamous cell carcinoma	0.998 ± 0.318	
Adenocarcinoma (-)	0.989 ± 0.318	0.001
Adenocarcinoma	1.165 ± 0.349	
Adenosquamous carcinoma (-)	1.023 ± 0.330	0.3097
Adenosquamous carcinoma	0.909 ± 0.309	

Values are presented as mean ± SD. ADC, DWI image-derived apparent diffusion coefficient.

(-): Indicates the absence of the pathological factor.

**Table 6:** ROC analysis of ADC values for assessing pathological factors in cervical cancer.

Pathological Factors	AUC	P Value	Optimal Cut-off*	Sensitivity	Specificity
Tumor diameter<4cm vs. ≥4cm	0.6529	0.0003	0.952	74.29%	49.21%
Well/Moderately vs. Poorly differentiation	0.6388	0.0038	0.939	68.29%	48.11%
Muscular layer infiltration<1/2 vs. >1/2	0.7282	<0.0001	0.953	75.16%	66.67%
Vaginal vault involvement	0.6008	0.0118	0.968	74.03%	47.28%
lymphovascular space involvement	0.6578	<0.0001	0.952	77.78%	54.94%
Adenocarcinoma	0.663	0.0016	0.978	66.67%	65.74%

*Tumor ADC (×10^-3^ mm^2^/s). ROC, receiver operating characteristic curve; AUC, area under the receiver operating characteristic curve; ADC, DWI image-derived apparent diffusion coefficient.

**Table 7** shows that tumor ADC<0.95×10^–3^ mm²/s was significantly associated with higher risk of tumor diameter ≥4 cm (OR, 2.60; 95% CI, 1.43–4.73), muscular layer infiltration >1/2 (OR, 5.46; 95% CI, 3.19–9.34), vaginal vault involvement (OR, 2.25; 95% CI, 1.28–3.96), and lymphovascular space involvement (OR, 3.81; 95% CI, 2.19–6.63). It was negatively associated with adenocarcinoma (OR, 0.31; 95% CI, 0.16–0.62).

**Table 7:** Associations between ADC value and pathological factors.

Pathological Factors	OR(95% CI)*	P Value
Tumor diameter≥4cm	2.60 (1.43-4.73)	0.0017
Poorly differentiation	1.82 (0.89-3.70)	0.0999
Muscular layer infiltration>1/2	5.46 (3.19-9.34)	<0.0001
Vaginal vault involvement	2.25 (1.28-3.96)	0.0051
Lymphovascular space involvement	3.81 (2.19-6.63)	<0.0001
Adenocarcinoma	0.31 (0.16-0.62)	0.0008

ADC, DWI image-derived apparent diffusion coefficient.

*OR is calculated to compare ADC<0.95×10^-3^mm^2^/s vs. ADC≥0.95×10^-3^mm^2^/s groups (ADC≥0.95×10^-3^ mm²/s as ref.) in predicting the outcomes (pathological factors).

## 4 Discussion

PLNM is a crucial factor in treatment planning and patient prognosis. Accurately determining the stage and identifying lymph node metastasis in a noninvasive manner is of significant importance. MRI is the most common radiological examination used for CC; however, the ability of conventional MRI to detect metastatic lymph nodes remains controversial. We examined the efficacy of the combination of conventional MRI and DWI functional imaging with ADC maps in PLNM diagnosis to define a better strategy to address the question. In this study, we analyzed 261 patients with CC who underwent surgery at our hospital. Conventional MRI combined with DWI correctly diagnosed 237 cases (38 PLNM, 199 non-PLNM CC), with 76% sensitivity, 94.31% specificity, 76% PPV, 94.31% NPV, and 90.8% accuracy. This performance was slightly better than that reported in previous studies (17–20). This might be because the diameter of short axis ≥10 mm was not the only criterion for identifying metastatic nodes in our study, and DWI with ADC improved the diagnostic accuracy. In our patients, the average lymph node diameter (short axis) of the PLNM group was 8.74 ± 5.13 mm. Therefore, a cut-off size of 10 mm was insufficient to effectively recognize metastatic nodes. Relying solely on the measurement of lymph node dimensions has certain limitations.

The average tumor ADC value in patients with PLNM was significantly lower than that in patients with non-PLNM CC. This was consistent with the result of a previous study by Song et al. (0.98 ± 0.12 vs. 1.070 ± 0.21×10^–3^ mm^2^/s) (21). After balancing the sensitivity (86%) and specificity (50%), the optimal cut-off of ADC=0.95×10^–3^mm^2^/s was obtained. Patients with a lower tumor ADC<0.95×10^–3^ mm^2^/s were associated with a 2.83–4 times higher risk of PLNM after adjusting for age and other risk factors. In the multivariate model 1, pathological factors (including tumor diameter, differentiation, muscular layer infiltration, and involvement) from the surgical pathology were included. Although the results may better reflect the actual situation, they may not be practical for use in clinical settings because these factors can only be confirmed after surgery. Therefore, we used the alternative factors available from MRI in model 2. An increase in tumor diameter (on MRI), vaginal vault involvement (on MRI), and tumor ADC<0.95×10^–3^ mm^2^/s were independent predictors of PLNM. We also found that tumor ADC<0.95×10^–3^ mm^2^/s was significantly associated with pathological risk factors, such as tumor diameter ≥4 cm, muscular layer infiltration >1/2, vaginal vault involvement, and lymphovascular space involvement. Decreased tumor ADC values can be an indicator of these pathological changes. A few previous studies have also found that ADC maps may distinguish between low- and high-grade CCs (22) or can be used to assess lymphovascular invasion (23) and parametrial invasion (24). Tumor ADC values may provide valuable insights into advanced-stage CC and prognostic information and can potentially aid in the risk stratification of disease. However, although the effect size of these relationships was considerable, the limitation of using ADC for detecting PLNM was its relatively low specificity, which was congruent with previous knowledge of the notable overlap in ADC values of benign and metastatic lymph nodes. In addition, the diagnostic efficacy of ADC in evaluating pathological factors is limited, as evidenced by the majority of AUC values falling between 0.6 and 0.7. Therefore, relying solely on ADC values is insufficient. Further investigations on the combined evaluation of ADC and other factors are required to better identify patients with PLNM.

The PLN groups in this study were categorized based on the widely recognized grouping of common iliac, external iliac, internal iliac, obturator, and deep femoral lymph nodes. The 5-year overall survival rate in patients can ranges from 20% to 85% (25). Survival prognosis is related to the number and sites of PLN metastases. Previous studies showed that the 5-year overall survival rates were 65.5%–85% if the number of positive PLN was 1–2 and 62.7% if the number of positive PLN was ≥3 (25–27). The 5-year overall survival rate in CC patients with metastasis to the common iliac lymph nodes was 20%–46.1%, which was worse than that in patients with metastasis to other lymph node regions (25, 28). Therefore, imaging evaluation of lymph node grouping and number is particularly important. The most common site of metastasis in CC is the obturator lymph nodes, followed by the internal, external, common iliac, and deep femoral lymph nodes. In the current study, the ranking of lymph node metastasis sites was as follows: obturator (30.39% of all metastatic lymph nodes), internal iliac (27.45%), external iliac (17.65%), common iliac (15.69%), and deep femoral (8.82%) lymph nodes, which is consistent with the results of previous studies and in accordance with lymph node metastasis pathways. When conventional MRI combined with DWI was used to evaluate each lymph node site, the sensitivity and PPV were generally lower than those of the overall diagnosis (except for the common iliac lymph nodes), whereas the specificity was very good at all sites. The ability of MRI and DWI to assess each lymph node site varies. There were difficulties for pelvic MRI in identifying the number of enlarged or metastatic PLNs owing to the signal similarity of the merged PLNs. We also analyzed the ADC values of the abnormal lymph nodes on MRI. There was an observable difference in the average lymph node ADC between patients with PLNM and those with non-PLNM (0.829×10^–3^ mm^2^/s vs. 1.119×10^–3^mm^2^/s). However, owing to the large range of values and value overlap, the difference was not statistically significant. Since normal lymph nodes are often small and difficult to detect, only patients with positive PLNM on MRI had available lymph node ADC data. Therefore, our results primarily represent patients with a high suspicion of PLNM. This factor also contributed to the suboptimal diagnostic performance of lymph node ADC in our study, because a significant number of true-negative results were not included. We anticipate future improvements in ADC construction capabilities to enhance resolution, thereby enabling the acquisition of negative data. More comprehensive data collection and analyses are required to examine the true association between lymph node ADC and PLNM.

This study has some limitations. First, constructing accurate ADC maps for normal lymph nodes is challenging, and our incomplete dataset of lymph node ADC values limits the generalizability of the results related to lymph node ADC. Consequently, we were unable to investigate further questions, such as the combined evaluation of lymph node size and ADC values. Second, the ROC analysis for lymph node ADC did not fully account for the clustered nature of our data, where multiple lymph nodes were assessed per patient. Third, this study was limited to newly diagnosed patients undergoing surgical resection, excluding those who received neoadjuvant chemotherapy and patients with advanced stages who were ineligible for surgery. This exclusion may have resulted in an underrepresentation of PLNM cases and an imbalance between the PLNM+ and PLNM- patient groups. Finally, DWI has limitations in differentiating between inflammatory lymph nodes, especially tuberculous and metastatic lymph nodes. The number of lymph nodes associated with prognosis could not be determined. Radiomics may be a solution to these issues; however, further studies with more comprehensive data are required.

## 5 Conclusion

Conventional MRI combined with DWI to detect PLNM showed good diagnostic performance, with 76% sensitivity, 94.31% specificity, and 90.8% accuracy. The tumor ADC value in patients with PLNM CC was significantly lower than that in patients with non-PLNM CC. Tumor ADC<0.95×10^–3^ mm^2^/s, increased tumor diameter on MRI, and vaginal vault involvement on MRI were independent predictors of PLNM. Tumor ADC<0.95×10^–3^ mm^2^/s was associated with a 2.83–4 times higher risk of PLNM than tumor ADC≥0.95×10^–3^ mm^2^/s. Tumor ADC<0.95×10^–3^ mm^2^/s was also associated with pathological factors, including tumor diameter ≥4 cm, muscular layer infiltration >1/2, vaginal vault involvement, and lymphovascular space involvement.

## Data availability statement

The raw data supporting the conclusions of this article will be made available by the authors, without undue reservation.

## Ethics statement

The studies involving humans were approved by ethics committee of Shenzhen People’s Hospital. The studies were conducted in accordance with the local legislation and institutional requirements. The ethics committee/institutional review board waived the requirement of written informed consent for participation from the participants or the participants’ legal guardians/next of kin because 1) It is a retrospective study with minimal risk to participants. 2) No adverse impact on participants’ rights and health. 3) Important value of the study. 4) Privacy and confidentiality were protected.

## Author contributions

LF: Formal analysis, Conceptualization, Investigation, Methodology, Writing – original draft. LM: Supervision, Validation, Writing – review & editing. RL: Data curation, Writing – original draft. XG: Data curation, Writing – original draft. HL: Writing – original draft, Investigation, Project administration. DY: Writing – original draft, Data curation, Visualization. ZL: Formal analysis, Software, Writing – review & editing.
